# Mechanism and Treatment of Right Ventricular Failure Due to Pulmonary Hypertension in Children

**DOI:** 10.3390/children12040476

**Published:** 2025-04-07

**Authors:** Bibhuti B. Das

**Affiliations:** Department of Pediatrics, Division of Pediatric Cardiology, University of Mississippi Medical Center, Jackson, MS 39216, USA; bbdas001@gmail.com; Tel.: +1-601-815-1432

**Keywords:** right ventricular failure, right heart failure, pulmonary hypertension

## Abstract

Pulmonary hypertension (PH) is a progressive disorder characterized by obstructive changes in the pulmonary vasculature, leading to increased pulmonary vascular resistance (PVR), right ventricular (RV) strain, and eventual RV failure (RVF). Despite advancements in medical therapy, PH remains associated with significant morbidity and mortality, particularly in children. RVF is a clinical syndrome resulting from complex structural and functional remodeling of the right heart, leading to inadequate pulmonary circulation, reduced cardiac output, and elevated venous pressure. Management paradigms for pediatric PH diverge significantly from those in adults, particularly due to the predominance of congenital heart disease (CHD) and the dynamic nature of pediatric cardiovascular and pulmonary development. CHD remains a principal driver of PH in children, and its associated pathophysiology demands a nuanced approach. In patients with unrepaired left-to-right shunts, elevated pulmonary blood flow can lead to progressive pulmonary vascular remodeling and increased PVR. The postoperative persistence or progression of PH may occur if irreversible vascular changes have already developed. Current PH treatments primarily focus on reducing PVR, yet distinguishing between therapeutic approaches that target the pulmonary vasculature and those aimed at improving RV function remain challenging. In pediatric patients with progressive PH despite optimal therapy, additional targeted interventions may be necessary to mitigate RV dysfunction and disease progression. This review provides a comprehensive analysis of the mechanisms underlying RVF in PH, incorporating insights from clinical studies in adults and experimental models, while highlighting the unique considerations in children. Furthermore, it explores current pharmacological and interventional treatment strategies, emphasizing the need for novel therapeutic approaches aimed at directly reversing RV remodeling. Given the complexities of RV adaptation in pediatric PH, further research into disease-modifying treatments and innovative interventions is crucial to improving long-term outcomes in affected children.

## 1. Introduction

The functionality of the right ventricle (RV) is a crucial factor in determining the prognosis for patients with pulmonary hypertension (PH). Once PH is established, the RV function begins to deteriorate, progressively worsening over time, and ultimately leading to RV failure (RVF) and premature death. Although this progression is well documented, the precise mechanisms driving RVF are not yet fully understood. Consequently, there are no approved treatments specifically designed to target the RV. This gap in targeted therapies can be attributed, in part, to the complex pathogenesis of RVF, as revealed in both animal models and clinical studies. The International Right Heart Foundation Working Group defines RVF as a “clinical syndrome caused by an alteration of structure and/or function of the right heart circulatory system that leads to suboptimal delivery of blood flow (high or low) to the pulmonary circulation and/or elevated venous pressures at rest or with exercise [1]”.

Guideline-directed medical therapy (GDMT) for managing left ventricular failure (LVF) includes angiotensin-converting enzyme inhibitors (ACEis), angiotensin II receptor blockers (ARBs), angiotensin receptor and neprilysin inhibitor (ARNI), sodium–glucose cotransporter-2 (SGLT2) inhibitors, β-blockers (BBs), and mineralocorticoid receptor antagonists (MRAs) [2,3]. However, ACEis/ARBs, BBs, and MRAs are not effective for patients with RVF due to PH unless they also have hypertension, coronary artery disease, or LVF [4]. Recent advancements in medical research have led to the development of innovative therapies, offering new hope for improving outcomes in patients with RVF in adults [5]. This review explores the mechanisms and novel pharmacological treatments of RVF due to PH, highlighting advancements from adult clinical trials and preclinical studies. While outcomes from adult trials cannot be directly extrapolated to children, the insights gained can help to understand the mechanisms behind RVF and to evaluate the potential effectiveness of therapeutic interventions in children.

## 2. Methodology

The goals for this review article was to summarize current treatment strategies for RVF in children with PH, to identify gaps in knowledge, and to suggest future research directions. A comprehensive literature search using databases such as PubMed, Embase, Scopus, and Cochrane Library was performed using a combination of keywords and medical subject headings (MeSH) terms, such as “pulmonary hypertension”, “right ventricular failure”, “pediatric” or “children”, “treatment” or “management”, “pharmacological therapy”, or “interventional therapy”. The inclusion criteria were a pediatric population, adult population, focus on RVF, clinical trials, preclinical studies, observational studies, and meta-analyses. The exclusion criteria were studies that did not meet the focus, e.g., non-English articles. The extracted data were organized into themes or categories, e.g., pathophysiology of RVF, differentiating pharmacological therapies targeting pulmonary vasculature versus RV adaptations, interventional strategies, long-term treatment outcomes, future directions, etc. The limitations of adult therapies when applied to children were discussed.

## 3. Definition and Hemodynamic Classification of PH

**Figure 1:**
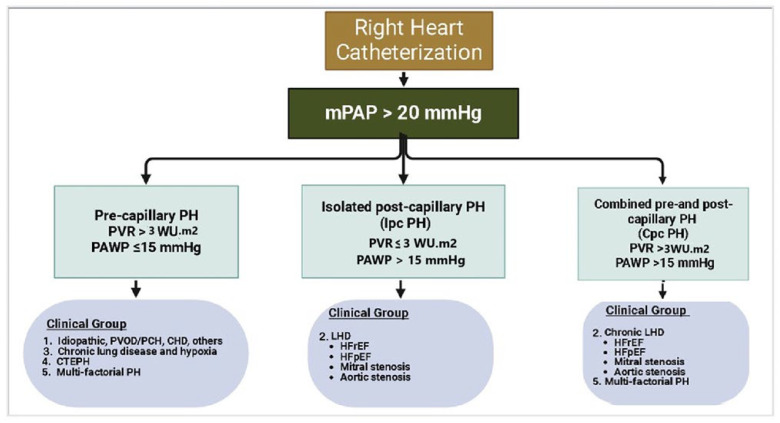
Types of pulmonary hypertension based on hemodynamics (original diagram) [IpcPH, isolated postcapillary pulmonary hypertension; CpcPH, combined postcapillary and precapillary PH; PVOD/PCH, pulmonary vaso-occlusive disease/pulmonary capillary hemangiomatosis].

The clinical definition and classification of PH are the same for both children and adults. In 2018, the definition of PH was updated to an elevation of the mean pulmonary artery pressure (mPAP) > 20 mm Hg at sea level in children older than 3 months [6]. However, the pulmonary vascular resistance (PVR) criterion of ≥3 Woods Unit (WU) per m^2^ in children remained unchanged [7]. PH, whether precapillary, isolated postcapillary (IpcPH), or combined postcapillary and precapillary PH [CpcPH]), increases RV afterload, leading to hypertrophy and eventual RVF [8]. Pulmonary arterial hypertension (PAH) is hemodynamically characterized by precapillary PH in the absence of other causes of precapillary PH, such as chronic thromboembolic pulmonary hypertension (CTEPH) and PH associated with lung diseases, and is characterized by a PVR of >3 WU·m^2^ and a pulmonary arterial wedge pressure (PAWP) of 15 mm Hg or less [6]. IpcPH is hemodynamically defined as mPAP > 20 mmHg, PAWP > 15 mmHg, and PVR ≤ 3 WU·m^2^. PVR is used to differentiate between patients with postcapillary PH who have a significant precapillary component (PVR > 3 WU·m^2^ combined with postcapillary and precapillary PH [CpcPH]) and those who do not (PVR ≤ 3 WU·m^2^—isolated postcapillary PH [IpcPH]) [6]. The differentiation of PH into pre-, post-, and combined types based on mPAP and PVR by right heart catheterization is described in Figure 1.

In the pediatric population, PH differs markedly from the adult counterpart in terms of classification, etiology, clinical presentation, and therapeutic strategies (Table 1).

The pediatric PH landscape is characterized by unique pathophysiological mechanisms and age-specific considerations that necessitate a tailored, multidisciplinary approach. Among the World Health Organization (WHO) PH classifications, Group 1 PH—comprising idiopathic pulmonary arterial hypertension (iPAH), heritable forms (HPAH), and congenital heart disease-associated PAH (CHD-PAH), including Eisenmenger syndrome—predominates in the pediatric population. In contrast, Group 2 PH, typically driven by left heart disease in adults, is rare in children due to the absence of acquired comorbidities such as diastolic dysfunction, left ventricular hypertrophy, or ischemic cardiomyopathy. When structural left-sided lesions are present in children, they are often corrected early or reclassified under CHD-PAH. Group 3 PH, secondary to developmental lung diseases such as bronchopulmonary dysplasia (BPD), congenital diaphragmatic hernia (CDH), or alveolar capillary dysplasia, is also common in infancy and early childhood, while Groups 4 (chronic thromboembolic PH) and 5 (multifactorial or unclear mechanisms) are rarely encountered and are managed in a case-specific manner. Management paradigms for pediatric PH diverge significantly from those in adults, particularly due to the predominance of CHD and the dynamic nature of pediatric cardiovascular and pulmonary development. CHD remains a principal driver of PH in children, and its associated pathophysiology demands a nuanced approach. In advanced or irreversible cases, therapies that target both RV and pulmonary vascular remodeling should be central to management strategies. This review focuses on reversing RV remodeling and addressing PH broadly, rather than concentrating on specific etiologies.

## 4. Pathophysiology of RVF Due to PH

**Figure 2:**
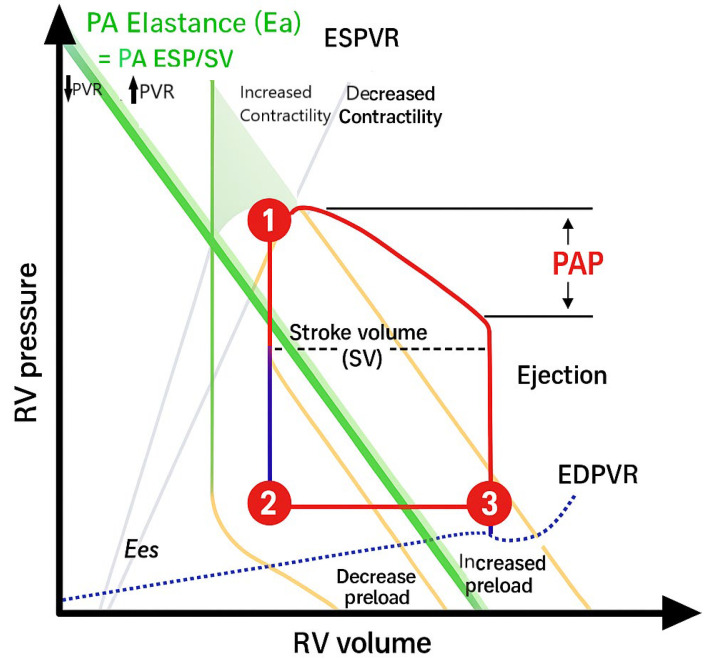
Pressure—volume loop of the RV (original diagram) [ESP, end-systolic pressure; EDP, end-diastolic pressure; ESV, end-systolic volume; EDV, end-diastolic volume; ESPVR, end-systolic pressure—volume relationship; EDPVR, end-diastolic pressure—volume relationship; Ea, arterial elastance; Ees, ventricular elastance].

The stroke work (SW) of the LV and RV differs due to several differences in the embryological, morphological, and myocardial characteristics of the RV and LV, as well as differences in the mean arterial pressure (MAP) and mPAP [9]. RV SW is calculated as the product of stroke volume (SV) and the transpulmonary gradient, and it is a measure of the RV’s efficiency in pumping blood [10]. RV elastance (Ees) is calculated from the slope of the end-systolic pressure–volume relationship (Figure 2). The elastance of the PA (Ea) is a measure of the total afterload faced by the RV and depends upon PVR and compliance. The maximal transfer of mechanical SW from the RV to the PA occurs when Ees ≥ Ea (RV–PA coupling) is present [11].

**Figure 3:**
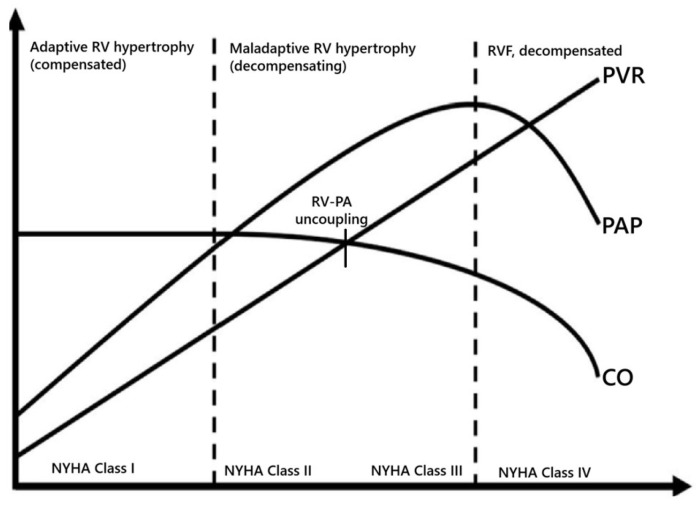
Hemodynamic progression in pulmonary hypertension (original diagram) [PAP, pulmonary artery pressure; PVR, pulmonary vascular resistance; CO, cardiac output; NYHA, New York Hearth Association; RV-PA, right ventricle—pulmonary artery].

Persistent pulmonary vascular remodeling in PH is driven by a combination of vasoconstriction and the excessive proliferation of pulmonary artery smooth muscle cells (PASMCs). Initially, the RV adapts to increased afterload through compensatory mechanisms such as hypertrophy and enhanced contractility to maintain RV-PA coupling. However, as PH progresses, RV-PA uncoupling (Ea > Ees) occurs, which is characterized by RV dilation and increased wall stress and is a sign of impending RVF [12]. The progression to RVF is marked by a decline in cardiac output (CO) and mPAP, as the RV can no longer sustain the increased afterload (Figure 3). This hemodynamic deterioration often leads to systemic congestion and end-organ dysfunction with New York Hearth Association (NYHA) functional class III to IV. In patients with PH, RVF is the primary cause of mortality, underscoring the importance of understanding its pathophysiology. Recent studies have highlighted the role of advanced hemodynamic phenotyping and machine learning in identifying distinct RV sub phenotypes, which may guide personalized therapeutic strategies [13]. For example, cluster analysis has revealed subgroups of PH patients with varying degrees of RV-PA coupling, diastolic stiffness, and afterload, providing insights into the transition from adaptive to maladaptive remodeling.

Furthermore, a chronic elevation in afterload triggers metabolic reprogramming in the RV, shifting its primary energy source from fatty acid oxidation (FAO) to glycolysis [14]. This shift is mediated by the downregulation of genes associated with oxidative phosphorylation and other key metabolic regulators, such as peroxisome proliferator-activated receptor alpha (PPARα) and peroxisome gamma coactivator-one alpha (PGC-1α), which are critical for energy metabolism and mitochondrial biogenesis [15]. Over time, this metabolic adaptation leads to increased RV mass and microvascular ischemia, driven by a mismatch between oxygen (O_2_) supply and demand. The RV reverts to a fetal-like metabolic profile, characterized by reduced oxidative capacity and diminished ATP production, resulting in energy deficiency and the loss of metabolic flexibility [16].

**Figure 4:**
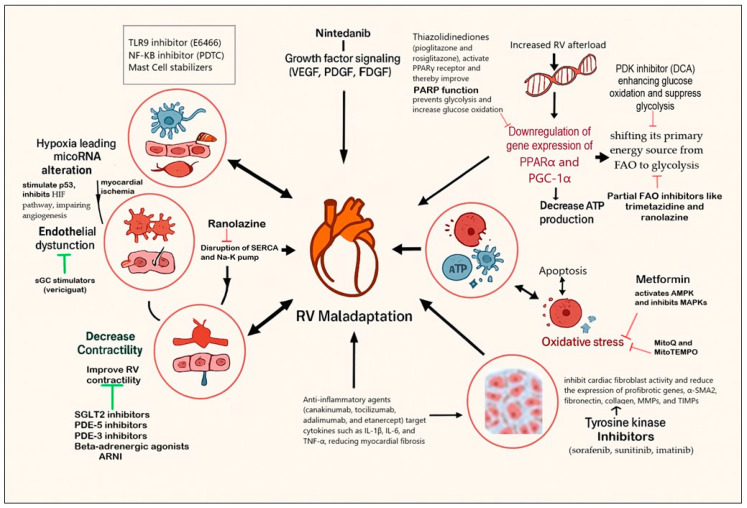
Experimental therapies for right ventricular failure (RVF) targeting novel pathways and therapeutic strategies (original diagram created for illustrative purposes). [Green arrows indicate pathway activation or stimulation. Red lines denote pathway inhibition. Note: Some pathways have been simplified for clarity and visual representation. HIF, hypoxia inducible factor; sGC, soluble guanylate stimulator; PDE5, phosphodiesterase 5; ECM, extracellular matrix; TLR9, toll-like receptor 9 (TLR9); NF-kB, nuclear factor kappa-light-chain-enhancer of activated B cells; SERCA, sarcoplasmic/endoplasmic reticulum calcium ATPase; ARNI, angiotensin neprilysin inhibitors; SGLT2i, sodium–glucose cotransporter 2 inhibitors; PDE3, phosphodiesterase 3; VEGF, vascular endothelial growth factor; PDGF, platelet-derived growth factor; FDGF, fibroblast growth factor; AMPK, 5′-adenosine monophosphate-activated protein kinase; MAPK, mitogen-activated protein kinase; p53, tumor protein 53; PGC-1α, peroxisome gamma coactivator-one alpha (PGC-1α); PARP, poly (ADP–ribose) polymerase; PPAR, peroxisome proliferator activated receptor; ATP, adenosine triphosphate].

The pathogenesis of maladaptive RV remodeling involves a complex interplay of multiple processes and signaling pathways (Figure 4). These include inflammation [17], fibrosis [18], impaired calcium (Ca^2+^) homeostasis [19], endothelial dysfunction, impaired nitric oxide (NO) synthesis [20], and the dysregulation of growth factor signaling pathways such as transforming growth factor-β (TGF-β) and impaired angiogenesis [21]. These interactions exacerbate the production of reactive oxygen species (ROS), which open mitochondrial permeability transition pores, inducing apoptosis by promoting fission, inhibiting fusion, and impairing mitochondrial metabolism and mitochondrial dynamics [22]. These changes lead to mitochondrial fragmentation and the disruption of myocardial energetics, further contributing to RV dysfunction.

## 5. Experimental Therapies Targeting RV Remodeling

Several experimental therapies have shown promise in reversing maladaptive RV changes (Figure 4). For instance, the pyruvate dehydrogenase kinase (PDK) inhibitor dichloroacetate (DCA) has been effective in preclinical models by enhancing glucose oxidation and suppressing glycolysis, thereby improving RV function and restoring mitochondrial dynamics [23]. Partial FAO inhibitors like trimetazidine and ranolazine have also demonstrated benefits by increasing glucose oxidation and improving RV function in models of RV hypertrophy [24]. Ranolazine, in particular, blocks the late Na^+^ current, reducing cytosolic Ca^2+^ levels and improving cardiac energy efficiency [25]. In a multicenter study, ranolazine improved RV function, LV end-diastolic volume, and biventricular stroke volume [26]. Thiazolidinediones, such as pioglitazone and rosiglitazone, activate peroxisome proliferator-activated receptor gamma (PPARγ), a key regulator of glucose and lipid metabolism, including mitochondrial FAO [27]. These agents normalize cardiac energy metabolism and have shown potential in mitigating RV dysfunction [28]. Antioxidants, including coenzyme Q, have been effective in reducing ROS production and improving mitochondrial function in animal models of PH [29].

Metformin, a widely used antidiabetic drug, has shown promise in reducing RV hypertrophy and improving diastolic function through the activation of 5′-adenosine monophosphate-activated protein kinase (AMPK) and the inhibition of mitogen-activated protein kinases (MAPKs) [30,31]. By reducing oxidative stress and preventing fibrosis, metformin inhibits key contributors to RV hypertrophy [32].

RV maladaptation is closely linked to inflammation and fibrosis. Multikinase inhibitors like nintedanib and sorafenib target vascular endothelial growth factor receptors (VEGFRs), platelet-derived growth factor receptors (PDGFRs), and fibroblast growth factor receptors (FGFRs) [33]. These agents inhibit cardiac fibroblast activity and reduce the expression of profibrotic genes, such as alpha-smooth muscle actine 2 (α-SMA2), fibronectin, collagen, matrix metalloproteinases (MMPs), and tissue inhibitors of metalloproteinases (TIMPs), thereby improving RV function in preclinical models. Mast cell activation, for example, releases inflammatory mediators that exacerbate RV remodeling [34]. Inhibitors such as cromolyn sodium and montelukast have been proposed to block mast cell activation, thereby attenuating inflammation [35]. Anti-inflammatory agents like canakinumab, tocilizumab, adalimumab, and etanercept target cytokines such as IL-1β, IL-6, and TNF-α, reducing myocardial fibrosis in preclinical models of RV remodeling [36,37].

Hypoxia-inducible factor (HIF) plays a critical role in promoting angiogenesis via VEGF, ensuring adequate coronary flow and O_2_ supply [38]. However, in advanced RV hypertrophy, severe hypoxia and microRNA induction exacerbate ROS production, activating the tumor suppressor protein p53 [39]. This inhibits the HIF pathway, impairing angiogenesis and further stimulating ROS production. Mitochondrion-targeted antioxidants like MitoQ and MitoTEMPO have shown potential in mitigating oxidative damage and inflammation, reversing RV remodeling in experimental models [40]. Stem cell therapies with proangiogenic properties represent a novel approach to improving RV function [41]. These therapies aim to enhance coronary flow and O_2_ supply, addressing the underlying metabolic and structural deficits in RVF.

Several emerging therapies are being investigated for their potential to reverse RV remodeling in children. Stem cells release extracellular vesicles (EVs), such as exosomes, containing bioactive molecules that can influence recipient cell behavior, offering a cell-free therapeutic strategy for PH. For example, EVs derived from neonatal heart tissues have shown strong pro-proliferative, anti-apoptotic, and proangiogenic activities in preclinical models [42]. These EVs, particularly those from regenerating neonatal tissues, have demonstrated the ability to promote cardiomyocyte proliferation and reduce fibrosis in experimental models of myocardial infarction, suggesting their potential applicability in pediatric RVF. While many of these therapies are still in preclinical or early clinical stages, their potential to address the unique pathophysiology of pediatric RVF is significant. Further research and clinical trials are needed to validate their efficacy and safety in children, but the current data provide a strong foundation for future therapeutic advancements.

## 6. Combination Therapy for RVF

ARNI, SGLT2 inhibitors, and soluble guanylate cyclase (sGC) stimulators represent promising therapeutic options for RVF due to their multifaceted mechanisms of action [43,44,45,46]. SGLT2 inhibitors, such as dapagliflozin, empagliflozin, canagliflozin, and sotagliflozin, promote natriuresis and glucosuria, leading to enhanced interstitial fluid loss compared to traditional diuretics [47]. Beyond their diuretic effects, these agents modulate nutrient signaling pathways, improve mitochondrial function, reduce oxidative stress, and attenuate inflammation and fibrosis, all of which are critical in RVF management [48,49,50,51,52,53]. ARNI, which combines valsartan (an angiotensin receptor blocker) and sacubitril (a neprilysin inhibitor), promotes vasodilation and diuresis by inhibiting angiotensin II type 1 receptors and increasing natriuretic peptide levels [54]. ARNI also exhibits antifibrotic and anti-inflammatory properties, which contribute to improved RV function [55,56,57,58,59]. Studies suggest that combining ARNI and SGLT2is is more effective in reducing PH and treating RVF [60,61]. SGC stimulators, such as riociguat and vericiguat, enhance cGMP production, which contributes to the maintenance of vascular tone and cardiac contractility while also reducing profibrotic and inflammatory pathways and counteracting myocyte hypertrophy [62,63]. The combined use of ARNI, SGLT2is, and sGC stimulators can maximize diuresis, reduce fluid retention, and decrease RV preload [64]. Their synergistic metabolic and anti-inflammatory effects further mitigate fibrosis, prevent apoptosis, and enhance myocardial energy efficiency by reversing adverse metabolic pathways and reducing ROS production [65]. Large-scale randomized clinical trials in adults have shown that none of the three drug classes led to a higher rate of treatment discontinuation due to adverse effects when compared to placebo [66]. However, additional research is needed to better understand the effectiveness of these medications across diverse patient groups, particularly in individuals with mid-range or preserved ejection fractions. Most clinical trials for ARNI, SGLT2is, and sGC stimulators have focused on adult populations, leaving a significant evidence gap for pediatric applications. Children have unique physiological and metabolic profiles, which may influence drug pharmacokinetics and pharmacodynamics. For instance, the immature renal and hepatic systems in younger children could alter the efficacy and safety of these therapies. Pediatric RVF often arises from congenital heart defects (CHD), which differ pathophysiologically from adult-onset RVF. These differences may limit the generalizability of adult findings to pediatric populations. The long-term effects of these therapies on growth, development, and organ function in children are unknown, necessitating cautious use and further research.

## 7. Current Treatment Approach to PH

**Figure 5:**
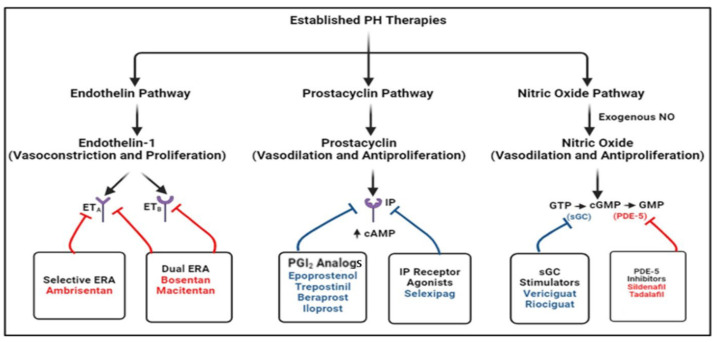
Mechanisms of action of established therapies for pulmonary hypertension (PH) targeting key signaling pathways (original diagram created for illustrative purposes). [Blue lines indicate pathway activation or stimulation. Red lines denote pathway inhibition. Note: Some pathways have been simplified for clarity and visual representation. ETA: endothelin receptor A; ETB: endothelin receptor B; ERA, endothelin receptor antagonist; cAMP: cyclic adenosine monophosphate; PGI_2_: prostaglandin I_2_; IP, prostacyclin receptor; NO: nitric oxide; sGC: soluble guanylate cyclase; PDE5: phosphodiesterase type 5; GTP: guanosine diphosphate; cGMP: cyclic guanosine monophosphate; GMP: guanosine monophosphate].

Pharmacological therapies for pediatric PH primarily target three major pathways: endothelin, nitric oxide, and prostacyclin (Figure 5). These agents have shown varying degrees of efficacy in improving RV function and overall outcomes. Phosphodiesterase-5 (PDE-5) inhibitors such as sildenafil, tadalafil, udenafil, vardenafil, and avanafil prevent PDE-5 degradation and enhance NO-mediated vasodilation by increasing cGMP. These agents have shown significant benefits in improving RV function and hemodynamics in pediatric PH patients [67,68,69,70]. Sildenafil is widely used due to its favorable safety profile and efficacy in reducing PVR. Endothelin receptor antagonists (ERAs), such as bosentan, ambrisentan, and macitentan, are commonly used to block the effects of endothelin, a potent vasoconstrictor. ERAs have demonstrated efficacy in reducing PVR and improving exercise capacity in children with PH [71,72,73,74]. However, their use is often limited by their side effects, such as liver toxicity and fluid retention. Prostacyclin receptor (IP) analogs, such as epoprostenol, treprostinil, iloprost, and beraprost, and the IP receptor agonists selexipag and ralinepag, have been shown to decrease PVR and improve functional capacity in clinical trials [75,76,77]. These drugs are potent vasodilators that also inhibit platelet aggregation. These agents are particularly effective in severe PH cases, improving RV function and survival. However, their administration routes (intravenous or subcutaneous) can be challenging in pediatric patients. Recently, treprostinil inhalation powder has become available, offering effective once-daily dosing superior to that of beraprost [78]. Despite advancements, the long-term outcomes remain suboptimal for many children with PH. Factors such as disease severity, underlying etiology, and delayed diagnosis can impact prognosis. A recent study found that adverse event-free survival rates for children with PH remain suboptimal, highlighting the need for improved management strategies [79]. Effective risk stratification is crucial for optimizing personalized treatment approaches. The key independent predictors of both early- and long-term adverse outcomes that have been identified are age, etiology, WHO functional class, and PVR, and these serve as a foundation for the ongoing development and refinement of treatment strategies and guidelines in pediatric PH.

The pharmacologic management of pediatric PH is highly individualized and is guided by disease classification, severity, vasoreactivity testing, and developmental considerations. PDE5 inhibitors, such as sildenafil, are considered a first-line therapy in Group 1 PH. ERAs, such as bosentan, are also widely utilized, but necessitate regular hepatic monitoring due to the risk of hepatotoxicity. Prostacyclin analogs—including intravenous epoprostenol and subcutaneous or inhaled treprostinil—are reserved for advanced or refractory cases, particularly in Eisenmenger physiology or rapidly progressive disease. Inhaled nitric oxide (iNO) plays a critical role in acute neonatal PH (persistent PH of newborns) and perioperative management for transient pulmonary vasodilation. Calcium channel blockers are only indicated in a minority of patients who demonstrate positive vasoreactivity during right heart catheterization.

**Figure 6:**
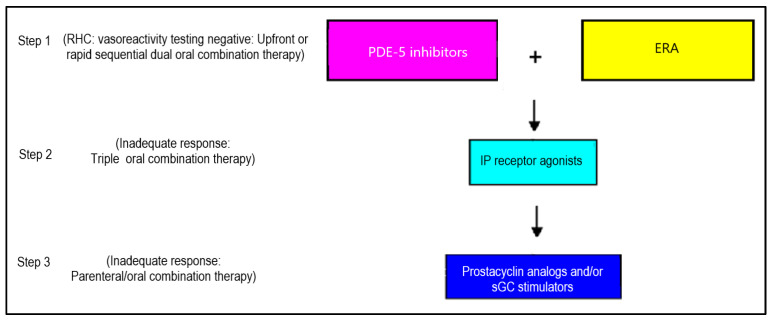
Proposed sequencing of add-on therapy for pediatric patients with PH [RHC: right heart catheterization; PDE-5: phosphodiesterase-5; ERA: endothelial receptor antagonists; IP: prostacyclin receptor; sGC: soluble guanylate cyclase].

Pediatric PH shares similarities with the adult form of the disease but also involves the additional issues of pediatric-specific lung and heart disorders that necessitate unique approaches. Due to the numerous challenges in conducting multicenter randomized controlled trials in children with PH and the lack of robust scientific evidence on efficacy and safety, regulatory authorities have not approved all PH medications for pediatric use. As a result, the current pharmacological management of pediatric PH relies primarily on real-world data, expert consensus statements on the off-label use of PH treatments, and extrapolated data from large adult randomized controlled studies. The European Society of Cardiology (ESC)/European Respiratory Society (ERS) guidelines endorse the use of adult-designed treatment algorithms for children, demonstrating the superiority of combination therapy over monotherapy [6]. Compared to monotherapy, targeted drug combinations for PH significantly enhance exercise tolerance, improve pulmonary hemodynamic parameters, and decrease the risk of serious adverse events and clinical deterioration in patients [80,81,82]. The combinations of bosentan with sildenafil and bosentan with iloprost show notable efficacy and a better safety profile than monotherapy for PH treatment [83]. Additionally, the combination of sildenafil with epoprostenol exhibits a low risk of clinical worsening in PH [83]. The proposed tailored strategy for choosing pharmacological therapy for pediatric PH should be based on the patient’s hemodynamic profile, vasoreactivity testing during right heart catheterization, and RV function (Figure 6). However, prior studies were performed when the diagnostic criterion for PH was an mPAP of 25 mmHg by right heart catheterization. Therefore, new randomized clinical studies are needed to know which drug combinations are effective and which drug combinations are ineffective in pediatric PH.

## 8. Disease-Modifying Agents Based on Preclinical and Clinical Trials

**Figure 7:**
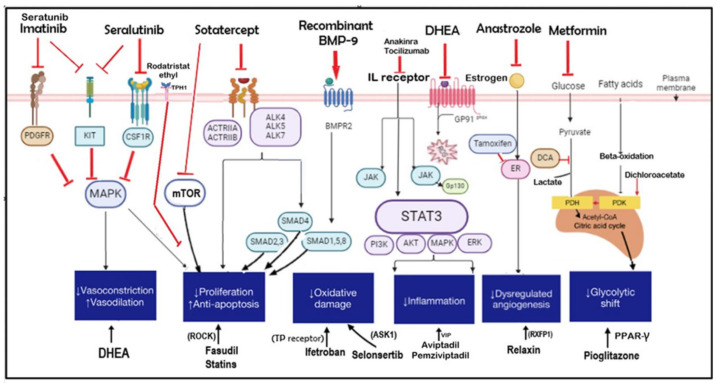
Mechanisms of selected novel therapies for the treatment of PH which also target RV remodeling (original diagram). [The green line indicates pathway stimulation; the red line indicates inhibition. Some pathways have been simplified for the purposes of depiction within the diagram. KIT, CD117 or c-lit; ASK1, apoptosis signal-regulating kinase 1; JAK, Janus kinase; BMP 9, bone morphogenetic protein 9; mTOR, mammalian target of rapamycin; MAPK, mitogen-activated protein kinase; PARP, poly (ADP–ribose) polymerase; PDH, pyruvate dehydrogenase; PDGFR, platelet-derived growth factor receptor; PDK, pyruvate dehydrogenase kinase; PPAR-γ, peroxisome proliferator-activated receptor gamma; ROCK, Rho-associated protein kinase; ACTRII, activin receptor type II; DHEA, dehydroepiandrosterone; ALK, activin receptor-like kinase; CSF1R, colony-stimulating factor 1 receptor; SMAD, SMA- and MAD-related proteins; STAT3, signal transducer and activator of transcription; TP, thromboxane-prostanoid receptor].

Current treatments for PH primarily focus on pulmonary vasodilation by addressing imbalances in vasoactive factors. Differentiating between pulmonary vascular and RV-targeted treatments requires a comprehensive approach that integrates experimental models (PAB), imaging techniques (such as echocardiography and cardiac MRI for assessing RV function), and biomarker analysis (NT-proBNP) [6]. While pulmonary vascular therapies work to lower PVR, RV-targeted treatments aim to enhance RV function and prevent failure. Recent studies have uncovered new disease pathways related to cell proliferation, metabolism, and inflammation, leading to emerging therapeutic possibilities (Figure 7).

Tyrosine kinase inhibitors like imatinib and seralutinib suppress tyrosine kinase activity through CD117 (KIT), PDGFR, CSF1R, and the MAPK pathway, thereby limiting PASMC proliferation [84,85,86]. Sotatercept, a novel fusion protein, combines the extracellular domain of activin receptor type IIA (ACTRIIA) with the Fc domain of human IgG1. It acts as a ligand trap, regulating SMAD proteins within the TGFβ superfamily, balancing growth-promoting and growth-inhibiting pathways (ACTRIIA and BMPR2, respectively) [87]. Additionally, sotatercept reduces PASMC hyperplasia by blocking mTOR, with promising outcomes confirmed in the phase 3 STELLAR trial, which showed significant improvements in the six-minute walk test and other clinical measures compared to a placebo [88].

Variants in BMP9 have been linked to PH, and some patients have exhibited reduced BMP9 expression even without these variants [89]. Recombinant BMP9 (rBMP9) mimics natural BMP9 signaling and has demonstrated the ability to reverse pulmonary vascular remodeling in severe PH models [90]. Therapies targeting sex hormones, such as anastrozole, tamoxifen, and dehydroepiandrosterone (DHEA), are under investigation for PH treatment [91,92].

The metabolic modulator DCA triggers PASMC apoptosis, helping to prevent and reverse chronic hypoxic PH in animal studies [93]. Anti-inflammatory agents, including anakinra, sarilumab, and siltuximab, modulate immune responses by targeting cytokines like IL-1 and IL-6 [94]. These cytokines activate the JAK-STAT pathway, leading to STAT3 translocation and the regulation of genes involved in PASMC proliferation, apoptosis, and inflammation [95]. Relaxin, a hormone, binds to RXFP1, influencing angiogenesis, reducing blood pressure, and improving cardiac output [96].

Rho-associated protein kinase (ROCK) inhibitors play a key role in vasoconstriction, vascular reactivity, and PASMC proliferation in animal PH models. The ROCK inhibitor fasudil has been shown to reduce pulmonary artery pressure in rats [97]. Combining rosuvastatin with fasudil resulted in greater reductions in RV pressure and hypertrophy compared to fasudil alone [98]. Ifetroban, a selective thromboxane receptor (TP) antagonist, has anti-inflammatory, vasodilatory, and anti-platelet aggregation effects [99]. Selonsertib, originally developed for liver failure, inhibits apoptosis signal-regulating kinase 1 (ASK1), thereby reducing inflammation, apoptosis, and fibrosis, making it a potential PH treatment [100].

Serotonin, a potent pulmonary vasoconstrictor and angiogenic agent synthesized from L-tryptophan via tryptophan hydroxylase (TPH) and metabolized by monoamine oxidase (MAO), is upregulated in the pulmonary arterial endothelial cells (PAECs) of patients with PH, where it acts in a paracrine manner on PASMCs to promote proliferation, contraction, and increased vascular tone [101,102]. Rodatristat ethyl, a TPH 1 inhibitor, reduces serotonin release and has undergone clinical trials for PH [103]. VIP analogs such as pemziviptadil and aviptadil have also shown promise in preclinical PH studies [104]. Further research is needed to assess the safety, effectiveness, and tolerability of serotonin antagonists and VIP analogs for PH treatment.

MicroRNAs (miRs) are small non-coding RNA molecules that regulate gene expression by binding to mRNA and inhibiting translation, playing a crucial role in disease processes, including PH [105]. In PH, miR-21 has shown variable expression patterns, being downregulated in some preclinical models while being upregulated in hypoxic conditions, where it contributes to pulmonary vascular remodeling by targeting BMPR2 [106,107]. Additionally, miR-124 is downregulated in PH, facilitating fibroblast activation and PASMC dysfunction, making miRs potential therapeutic targets for PH management [108].

## 9. Interventions for Children with Advanced Pulmonary Hypertension

Beyond pharmacologic intervention, comprehensive long-term management necessitates a multidisciplinary approach that addresses the full spectrum of pediatric development. Care extends beyond pulmonary hemodynamics to include somatic growth, neurocognitive outcomes, exercise capacity, and psychosocial well-being. Regular surveillance and reassessment are imperative given the evolving nature of cardiovascular and pulmonary physiology in children. Specialized pediatric PH centers facilitate individualized care plans, coordinate transition to adult services, and offer genetic evaluation and counseling when indicated. Surgical and interventional decision-making in pediatric PH requires careful consideration of operability, particularly in CHD-PAH. The early repair of congenital defects is critical to prevent irreversible pulmonary vascular disease; however, operability assessments must rely on invasive hemodynamic data and vasoreactivity testing. In selected patients with borderline PVR elevation, a “treat-and-repair” strategy can be used—initiating targeted PAH therapy preoperatively to optimize pulmonary vascular hemodynamics prior to surgical repairs under active investigation, though its long-term efficacy remains uncertain. Patients with Fontan circulation represent a distinct subgroup in whom pulmonary vascular pathology deviates from conventional PH classifications, necessitating specialized surveillance strategies.

There are no standardized guidelines for determining which patients who have not responded to conventional medical therapy should undergo atrial septostomy, a pulmonary-to-systemic shunt, lung transplantation, or a combination of these procedures. Generally, a multidisciplinary team should assess each patient individually to decide on the most appropriate approach. This evaluation should consider risk factors, technical feasibility, available medical expertise, and the optimal sequence of interventions.

### 9.1. Creation of Atrial-Level Communication

Creating a shunt at the atrial level can enhance LV preload and CO, but it comes at the cost of reduced systemic oxyhemoglobin saturation. The risks associated with atrial-level communication are higher in patients with significantly elevated right-atrial pressure, pre-existing cyanosis, severe parenchymal lung disease, or an increased likelihood of thromboembolic events. Notably, atrial-level intervention for a failing RV offers volume relief rather than pressure relief, as it does not directly lower RV afterload. Recently, transcatheter atrial shunt therapies including an atrial flow regulator-type device, designed to prevent recurrent syncope, have been developed, which are safe and easily placed in children with acute RVF [109,110,111]. The American Thoracic Society suggests atrial-level shunt intervention for selected children with progressive PH and RVF despite undergoing optimal therapy (conditional recommendation, very low certainty of evidence) [112].

### 9.2. Pulmonary-to-Systemic Shunt (Reverse Potts Shunt)

Like atrial-level communication, creating a Potts shunt through a transcutaneous or surgical approach can decrease preloading of the RV and enhance overall CO, although it results in decreased systemic oxyhemoglobin saturation distal to the shunt. Unlike atrial-level communication, this procedure maintains normally saturated blood flow to the cerebral and coronary arteries and significantly reduces RV afterload. The risk associated with a Potts shunt is higher in patients with RVF, and those requiring ECMO support, ICU admission, or mechanical ventilation. The approach to Potts shunt creation has evolved over time, now incorporating the use of unidirectional valved shunts in patients with suprasystemic PH [113]. When patient selection is performed thoughtfully and the procedure is performed at a center with significant experience in both the creation of a Potts shunt and the management of advanced pediatric PH, early mortality may be reduced, and patients can be bridged successfully to lung transplantation.

### 9.3. Lung Transplantation

Atrial or systemic shunts are often considered palliative options for children with advanced PH, while lung transplantation is seen as a more definitive treatment due to the normal pulmonary vasculature of the allograft. If a patient is not eligible for a Potts shunt or the creation of atrial level communication, despite receiving optimal medical treatment, an immediate evaluation for transplantation should be considered. Additionally, it is important to be familiar with the policies of the local transplant center to determine the feasibility of lung transplantation following a Potts shunt. Pediatric lung transplantation carries inherent risks and has a 5-year survival rate of 50–60% [114]. Recently, improvements in lung transplant outcomes have been observed, along with the recovery of RV function post-transplant [115,116]. Combined heart–lung transplantation for PH is reserved for rare cases involving uncorrectable CHD, co-existing LV dysfunction, and technical issues such as significant right heart enlargement in young children, donor lung constraints, and the risk of airway caliber compromise with tracheal versus bi-bronchial anastomoses in small children.

## 10. Future Directions

The therapeutic and pathophysiological framework of pediatric PH thus stands in contrast to adult paradigms, particularly given the rarity of Group 2 PH in children and the limited applicability of conventional HF pharmacotherapy such as ARNI, SGLT2i, MRA, and BBs. Treatment regimens are frequently off-label, dosed by body weight, and guided by individual clinical trajectory and underlying diagnosis. CHD-PAH remains the most clinically relevant and heavily researched category in children, with emerging therapeutics increasingly focusing on this population.

Although there has been significant progress in understanding the pathobiology of maladaptive RV dysfunction due to PH, improving the outcomes for children with PH remains a formidable challenge. This is largely due to the unique characteristics of pediatric-specific disorders and the scarcity of randomized clinical trial data to guide evidence-based therapeutic interventions. As a result, most RV-targeted drug therapies remain inadequately studied in children, with most of the current evidence extrapolated from adult clinical or preclinical trials. This gap in pediatric-specific data underscores the critical need for further research to validate novel therapies, particularly for infants and young children. The early detection of PH and the initiation of targeted therapies are critical for improving survival. Besides advances in echocardiographic and cardiac MRI, biomarkers are essential for the early diagnosis of PH. In addition to NT-pro-BNP (or BNP), several serum biomarkers have been identified in pediatric PH studies, including the insulin-like growth factor (IGF) [117], interleukin-6 [118], soluble suppressor of tumorigenicity (ST2) [119], and survivin [120], which play a significant role in PH disease progression and are reported to predict prognosis.

miRs have emerged as potential biomarkers for diseases involving impaired angiogenesis and may help to predict pediatric PH [121]. Circulating endothelial cells (CECs) and endothelial cell progenitors (ECPs) are detected in the blood of PH patients, with rising CEC levels preceding clinical deterioration in children with idiopathic PH and CHD-related PH [122]. Additionally, elevated CEC levels have been linked to disease irreversibility in CHD-related PH, highlighting their potential role in prognosis and monitoring treatment responses. These biomarkers should be validated in future pediatric PH studies.

There are several ongoing clinical trials of pediatric PH to evaluate the efficacy and safety of combination therapy and novel drugs in pediatric PH. A clinical trial of the sGC stimulator riociguat in children aged 6–17 years on stable ERA or prostacyclin therapy showed an increase in the 6 min walk distance and a decrease in NT-proBNP [123]. Additionally, a case series reported that young infants with neonatal PH were able to lower and stop iNO with riociguat due to its effective sGC stimulation. These data reveal a knowledge gap in the use of pulmonary vasodilators in neonatal PH [124]. There are several ongoing clinical trials aimed at providing more evidence-based data for treating children with PH. These include trials for macitentan (NCT02932410) and selexipag (NCT04175600), which are currently underway. Additionally, sotatercept, which restores balance in BMP pathways, is being tested in children (NCT05587712). Another key trial is a multicenter randomized controlled study by the Pediatric Pulmonary Hypertension Network, comparing mono and duo therapy in children at the time of PH diagnosis (NCT04039464).

Stem cell therapy has emerged as a promising approach for treating PH, aiming to address the underlying vascular remodeling and RV dysfunction characteristics of the disease. Stem/progenitor cells have demonstrated the ability to promote the endothelial repair of dysfunctional arteries and induce neovascularization, which are crucial in counteracting the endothelial dysfunction observed in PH [125]. Mesenchymal stem cells (MSCs) possess immunomodulatory properties that can attenuate inflammatory responses, thereby reducing pulmonary vascular remodeling and the progression of PH [126]. 

Tailoring therapies based on individual patient profiles, including genetic and molecular markers, could enhance the durability of the reverse remodeling of RV due to PH and long-term outcomes. Recent findings suggest that genetic factors in pediatric PH may influence outcomes and could be used in future risk stratification. For instance, children with a TBX4 mutation exhibited better survival rates than those with a BMPR2 mutation in one study [127]. While assessing the genetic causes of developmental lung disease offers valuable diagnostic and prognostic insights, the effects of individual variants on prognosis and treatment responses in childhood-onset PH remain unclear and need future exploration.

Low-intensity pulsed ultrasound (LIPUS) therapy has shown potential for activating endothelial nitric oxide synthase (eNOS) in various cardiovascular diseases [128]. In RVF due to PH, eNOS expression was downregulated [129]. In LIPUS-induced eNOS activation in two animal models of RVF, eNOS expression and its downstream pathways were improved [130]. These findings suggest that eNOS is a crucial therapeutic target for RVF, and LIPUS therapy presents a promising mechanical approach to enhancing RV function in PH patients.

Looking ahead, future directions in the management of RVF in children with PH should focus on several key areas. First, there is an urgent need for well-designed, pediatric-specific clinical trials to evaluate the safety and efficacy of emerging therapies. Collaborative efforts among researchers, clinicians, and regulatory bodies are essential to overcome the challenges of conducting trials in this population. Second, advancements in precision medicine, including the use of biomarkers and personalized treatment approaches, may enable more tailored and effective interventions. Finally, integrating pharmacological, mechanical, molecular, and regenerative therapies into a comprehensive treatment paradigm will be critical for addressing the multifactorial nature of RVF.

## 11. Conclusions

Among the investigational therapies, sotatercept—a novel fusion protein and activin signaling inhibitor that restores balance in the TGF-β/BMPR2 axis—has shown encouraging preliminary results in adult PAH and is now under evaluation in pediatric trials for iPAH and HPAH. The long-term prognosis in pediatric PH is multifactorial and hinges not only on hemodynamic parameters and the therapeutic response, but also on neurodevelopmental outcomes, comorbid conditions such as prematurity or genetic syndromes, and the quality of longitudinal multidisciplinary care. This underscores the need for an integrated, etiology-driven approach to pediatric PH that encompasses not only survival but holistic child health and development.

The balance between PH-specific therapies and RV-targeted interventions is pivotal in reversing maladaptive RV remodeling and improving clinical outcomes. RVF presents a complex clinical challenge due to its multifaceted pathophysiology and the limited availability of effective treatments. However, recent advancements in understanding the cellular and molecular mechanisms driving RVF have opened up new avenues for innovative therapeutic strategies. These include targeted molecular therapies, gene-based interventions, and regenerative approaches such as stem cell therapy, which hold promise for addressing the underlying causes of RV dysfunction. In conclusion, while significant challenges remain, the future of RVF management in children with PH is promising. By bridging the gap between preclinical discoveries and clinical applications, and by fostering collaboration across disciplines, we can pave the way for transformative therapies that improve the lives of children with this devastating condition.

## Figures and Tables

**Table 1:** Etiologies, Classification, and Incidence of Right Ventricular Failure in Children with PH by WHO Group.

	Group 1 (PAH: CHD-PAH, iPAH, HPAH)	Group 2 (PH Due to LHD)	Group 3 (PH Due to Lung Disease)	Group 4 (CTEPH)	Group 5 (Miscellaneous)
Common Pediatric Etiologies	CHD (e.g, ASD, VSD, AVSD), idiopathic PAH, BMPR2 mutations	Rare; congenital mitral/aortic lesions, cardiomyopathies	BPD, CDH, alveolar capillary dysplasia pediatric ILD	Exceptionally rare in pediatrics	Sarcoidosis, storage disorders, hematologic diseases
Incidence of RV Failure	High, especially in iPAH and Eisenmenger syndrome	Low to moderate, depending on LHD severity	Moderate to high in severe developmental lung disease	Variable, based on clot burden and RV adaptation	Variable
Medical Management	Targeted therapies (PDE5i, ERA, prostacyclins); CCB if vasoreactive; sotatercept in trials	Optimize LHD; PH therapies typically ineffective or harmful	Supportive care (oxygen, diuretics); PH therapies selectively in elevated PVR	Anticoagulation; surgical consideration if operable	Treat underlying cause; supportive care
Surgical/Intervent ional Role	CHD repair if operable; lung/heart-lung transplant in advanced disease	Valve repair/replacement; heart transplant in end-stage	Ventilatory support; transplant in refractory cases	Pulmonary thromboendarterectomy (rarely performed in children)	Individualized; etiology—dependent
Long-Term Prognosis	Variable; improved with advanced therapies; Eisenmenger may have stable course	Better if LHD is correctable; poor with progressive myocardial disease	Guarded; prognosis tied to underlying lung pathology	Unclear due to rarity	Heterogeneous outcomes

PAH: pulmonary arterial hypertension, CHD: congenital heart disease, HPAH: hereditary PAH, LHD: left heart disease, CTEPH: chronic thromboembolic pulmonary hypertension, PDE5i: phosphodiesterase 5 inhibitor, ERA: endothelin receptor antagonists.

## Abbreviations

RVD	Right ventricular dysfunction
RVF	Right ventricular failure
MAP	Mean arterial pressure
mPAP	Mean pulmonary artery pressure
PH	Pulmonary hypertension
ACEi	Angiotensin-converting enzyme inhibitors
ARBs	Angiotensin II receptor blockers
BBs	Beta-blockers
ARNI	Angiotensin receptor–neprilysin inhibitor
iNO	Inhaled nitric oxide
CO	Cardiac output
SW	Stroke work
SV	Stroke volume
RV-PA coupling	Right ventricle–pulmonary artery coupling
LIPUS	Low-intensity pulsed ultrasound
